# Design of human immunodeficiency virus-1 neutralizing peptides targeting CD4-binding site: An integrative computational biologics approach

**DOI:** 10.3389/fmed.2022.1036874

**Published:** 2022-11-18

**Authors:** Sandhya Vivekanandan, Umashankar Vetrivel, Luke Elizabeth Hanna

**Affiliations:** ^1^Department of Virology and Biotechnology, ICMR-National Institute for Research in Tuberculosis, Chennai, India; ^2^University of Madras, Chennai, India

**Keywords:** HIV-1, peptide therapeutics, CD4-binding site, neutralizing peptides, molecular dynamics simulation

## Abstract

Peptide therapeutics have recently gained momentum in antiviral therapy due to their increased potency and cost-effectiveness. Interaction of the HIV-1 envelope gp120 with the host CD4 receptor is a critical step for viral entry, and therefore the CD4-binding site (CD4bs) of gp120 is a potential hotspot for blocking HIV-1 infection. The present study aimed to design short peptides from well-characterized CD4bs targeting broadly neutralizing antibodies (bNAbs), which could be utilized as bNAb mimetics for viral neutralization. Co-crystallized structures of HIV-1 gp120 in complex with CD4bs-directed bNAbs were used to derive hexameric peptides using the Rosetta Peptiderive protocol. Based on empirical insights into co-crystallized structures, peptides derived from the heavy chain alone were considered. The peptides were docked with both HIV-1 subtype B and C gp120, and the stability of the peptide–antigen complexes was validated using extensive Molecular Dynamics (MD) simulations. Two peptides identified in the study demonstrated stable intermolecular interactions with SER365, GLY366, and GLY367 of the PHE43 cavity in the CD4 binding pocket, and with ASP368 of HIV-1 gp120, thereby mimicking the natural interaction between ASP368_gp120_ and ARG59_*CD*4–RECEPTOR_. Furthermore, the peptides featured favorable physico-chemical properties for virus neutralization suggesting that these peptides may be highly promising bNAb mimetic candidates that may be taken up for experimental validation.

## Graphical Abstract

Graphical flow chart of methodology. 
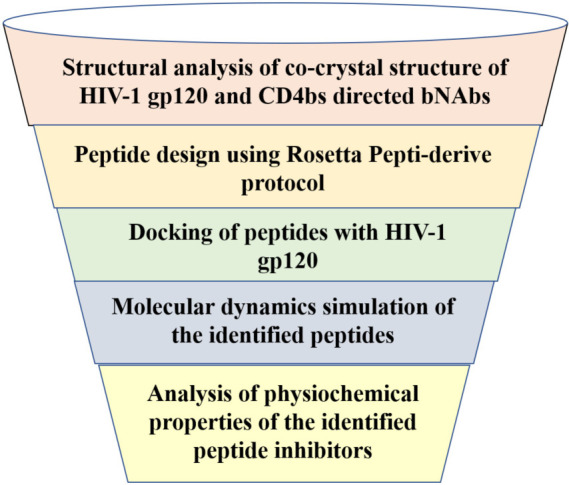

## Introduction

Human Immuno-deficiency Virus (HIV), the causative agent of Acquired Immuno-Deficiency Syndrome (AIDS), continues to be a tenacious global public health challenge. According to the UNAIDS 2021 report, there were 37.7 million people living with HIV (PLHIV), of which 27.5 million people were on Anti-Retroviral Treatment (ART) and 1.5 million people were newly infected with HIV in 2020^1^. Though 40 years have passed since the discovery of HIV, a preventive vaccine against HIV continues to be a dream of the future (1, 2). However, the introduction of combinatorial Anti-Retroviral Therapy (cART)/Highly Active ART (HAART) has revolutionized the treatment of HIV infection and contributed significantly to viral suppression in infected individuals and control of transmission (3, 4). However, the emergence of drug resistance and the establishment of long-lived latent reservoirs remain major obstacles to the cure of HIV infection and elimination of the disease (5, 6).

In recent years, broadly neutralizing antibodies (bNAbs) that can neutralize diverse HIV-1 strains by targeting vulnerable epitopes on the HIV-1 envelope and thereby block HIV-1 infection have gained attention as potential adjuncts to antiretroviral therapy (7, 8). Recent studies have demonstrated that the administration of bNAbs is effective in suppressing viremia (9) and protecting against lentiviral infection in animal models (10, 11), thus providing valuable insights for the design of effective HIV-1 vaccines (12, 13). Very recently, researchers have directed their attention towards the development of therapeutic proteins and peptides targeting HIV, due to their advantages such as specificity and selective nature of action as compared to drugs and antibodies (14–16). Enfuvirtide (also known as Fuzeon or T20), an FDA-approved peptide-based drug, prevents the completion of HIV fusion events and has been used in combination with other anti-retroviral drugs for treating HIV infection (17). However, the drug has limited clinical application due to the emergence of resistant HIV-1 strains (12, 18).

Selective interaction of the HIV-1 envelope glycoprotein (gp120) with the CD4 molecule which serves as the primary cellular receptor, and one of the chemokine receptors CCR5/CXCR4 or both, constitutes a crucial step in HIV-1 infection (19–21). Regardless of the genomic and antigenic variation between HIV-1 strains, the CD4 binding site (CD4bs) is known to be well-conserved among the different HIV-1 subtypes and is reported to be one of the potential targets of neutralizing antibodies (22–24). The CD4bs is centered in a cavity formed at the interface of the gp120 outer and inner domains, where the hydrophobic residues present in the deep pocket constitute the point of contact with Phe-43 of the CD4 receptor (also called the Phe43 cavity) (25, 26). In addition, Arg59 of the CD4 receptor forms a salt bridge with D368 of gp120 to stabilize the CD4 binding site interaction (27, 28).

As early as 1999, Vita et al. reported that oligo-peptides targeting the CD4bs could inhibit the binding of gp120 with the CD4 receptor and thereby prevent HIV infection (29). The present study is based on the hypothesis that short peptides derived from the paratope of broadly neutralizing antibodies might function as potent mimics of these antibodies. This is based on earlier reports that ultra-short peptides of size up to seven amino acids have several useful features including biocompatibility, tunability, non-immunogenicity, biodegradability, and most importantly, efficient survival against proteolytic degradation in the gastrointestinal tract, as compared to longer peptides (30). We chose ultra-short peptides of 6-amino acids length (hexamers) for our study. Taking advantage of the available HIV-1 gp120-neutralizing antibody crystal structure complexes, we made an attempt to identify hexameric peptides from the paratope of neutralizing antibodies and characterized them using *in silico* methods like Molecular modeling, interacting interface analysis, and Molecular Dynamic (MD) simulation to understand their usefulness as therapeutic tools for HIV.

## Materials and methods

### Selection of co-crystal structures of broadly neutralizing antibody with HIV-1 envelope gp120

**FIGURE 1:**
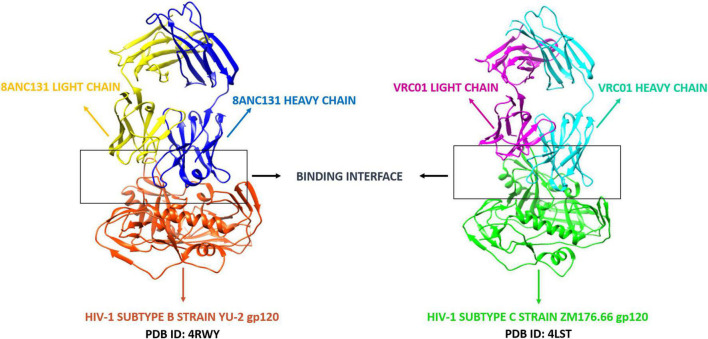
Co-crystal structures of 8ANC131-YU-2 gp120 and VRC01-ZM176.66 gp120. The secondary structure elements (alpha helix and beta sheets) color coded. PDB ID: 4RWY—Orange red: HIV-1 subtype B envelope, Blue: 8ANC131 Heavy chain, Yellow: 8ANC131 Light chain. PDB ID: 4LST—Green: HIV-1 subtype C envelope, Cyan: VRC01 Heavy chain, Magenta: VRC01 Light chain. The interface between the antibodies and HIV-1 gp120 are highlighted and shown as the binding interface/CD4-binding site. (These two antibodies were selected based on their neutralization profile and availability of crystal structure with HIV-1 subtype B and C envelope gp120 in Protein Data Bank).

A number of CD4bs-directed neutralizing antibodies have been identified and reported. Based on their mode of recognition and B-cell ontogeny, CD4bs antibodies fall into two categories: VH-gene restricted antibodies derived from the heavy chain germline genes VH1-2 or VH1-46, and CDRH3 dominated antibodies in which the antibody binding interfaces are dominated by the complementary-determining region three (CDR3) (13, 31, 32). The CD4bs directed bNAbs used for this study included VRC01 and 8ANC131, considered to be the first identified members of the VH-gene restricted “VRC01-class and 8ANC131-class” bNAbs (33) since the co-crystal structures of these antibodies with HIV-1B and C envelopes were available. VRC01 (VH1-2) and 8ANC131 (VH1-46) are both potent bNAbs found to be capable of neutralizing about 91 and 78% of the HIV-1 strains, respectively (34). The co-crystal structures of 8ANC131 with the HIV-1 subtype B envelope YU-2 gp120 (PDB ID: 4RWY 2.13 Å resolution) and VRC01 with the HIV-1 subtype C envelope ZM176.66 gp120 (PDB ID: 4LST 2.55 Å resolution) were downloaded from PDB (Protein Data Bank) (Figure 1). Both co-crystal structures included the heavy and light chains of the respective antibodies complexed with HIV-1 envelope gp120. The Fab (Fragment antigen-binding) regions of the antibodies were bound to the CD4bs in HIV-1 gp120.

### Design of short linear peptides targeting the CD4-binding site

The Rosetta Peptiderive is a computational tool designed to predict possible inhibitory peptides from the crystal structures of protein complexes based on their interacting interface, was used to identify short linear peptides that would target the CD4bs and bring about virus neutralization. This tool is hosted online in ROSIE (Rosetta Online Server that Includes Everyone) web interface and can be accessed at https://rosie.rosettacommons.org/peptiderive. The antigen (HIV-1 gp120)–antibody (bNAb) complex was uploaded on the Rosetta peptiderive tool in PDB format, with optimal parameters defining the Receptor and Partner. The tool automatically refines the antigen–antibody complex by removing local clashes and extracts potential peptide fragments of specified window size. The binding energies of the identified peptide–antigen complexes were calculated using the Rosetta energy function (35). Peptides with the most significant binding scores were shortlisted, and their position, sequence, interface score and relative interface score were obtained (36). Intermolecular interactions of the identified peptide-antigen complexes were visualized in the PDBsum webserver (37) and CHIMERA (38).

### Docking of peptides with human immunodeficiency virus-1 gp120

To validate the binding of the identified peptides with HIV-1 gp120, peptide–antigen docking was performed using HADDOCK (High Ambiguity Driven protein-protein DOCKing) webserver (Version 2.2) in the EASY interface available at https://wenmr.science.uu.nl/haddock2.4/. The antigen and peptides were docked by generating Ambiguous Interaction Restraints (AIR) with the interface residues identified from the PDBsum analysis of the Rosetta peptiderive complexes (39, 40). The docked structures were summarized in clusters, and each cluster was assigned a HADDOCK score, cluster size, RMSD from the overall lowest energy conformations, Z-score and buried surface area along with bonding energies (Vander Waal’s, electrostatic, desolvation, and restraints violation energies). The best-docked complex (topmost cluster suggested by HADDOCK) replicating the desired residual interactions was identified and selected for further analysis. The binding affinity (Δ*G*) and dissociation constant (*K*_*d*_) of the docked complexes were calculated using the PRODIGY webserver, available at https://wenmr.science.uu.nl/prodigy/. This webserver predicts binding affinities based on inter-molecular contacts within a distance cut-off of 5.5 Å (41, 42).

### Molecular dynamics simulations of the peptides with human immunodeficiency virus-1 envelopes

The peptide-HIV-1 envelope complexes identified using Rosetta peptiderive were subjected to Molecular dynamics (MD) simulations to deduce their dynamic behavior under physiologically simulated conditions (43). MD simulations were performed using the DESMOND software package (44) with OPLS_2005 as a force field and implemented as in Muthukumaran et al. (45). To begin with, the system was built in an auto-calculated cubic box and solvated with explicit Single Point Charge (SPC) water molecules. The solvated system was energy minimized and the MD run was carried out for 200 ns by implementing an NPT ensemble with a sampling interval of 10 ps. During the MD run, the whole system was maintained at an equilibrium of 300 K temperature and 1 atm pressure. Analytical tools available in DESMOND were used to infer the Root Mean Square Deviation (RMSD) of the protein backbone, the Root Mean Square Fluctuation (RMSF) of the residues, the radius of gyration, and other structural transitions throughout the simulations.

### Molecular mechanics-poisson boltzmann surface area calculation for the top-scoring stable neutralizing peptide-antigen complexes

The binding free energy (**Δ***G*) of the final frames of stable neutralizing peptide–antigen complexes obtained from the MD simulation was calculated by implementing MM-PBSA (Molecular Mechanics-Poisson Boltzmann Surface Area) protocol in farPPI (fast amber rescoring for Protein–Protein interaction Inhibitors) webserver, available at http://cadd.zju.edu.cn/farppi/. Precise binding energies of the docked poses were evaluated by the MM-PBSA method which combines energy calculations based on implicit solvent and molecular mechanics model (46). Among the MM-PBSA procedures, PB3 based approach was found to be highly accurate as compared to the other approaches in farPPI, as two force fields, GAFF2 and ff14SB, were applied to the peptide and antigen, respectively (47, 48). Hence, this method was adopted to score the binding free energy of the peptide–antigen complexes.

#### KDeep absolute binding affinity calculation for the most stable neutralizing peptide-antigen complexes

In addition to MM-PBSA, absolute binding affinity (Δ*G*) of the topmost neutralizing peptide–antigen complexes was calculated using KDeep, a protein–ligand affinity predictor tool available at https://playmolecule.com/Kdeep/. This predictor works based on a machine learning approach using a state-of-the-art 3D convolutional neural network (49). The input was voxelized into pharmacophore features like aromaticity, hydrophobicity, total excluded volume, etc., and passed onto the DCNN (Deep Convolutional Neural Network) model, which is pre-trained by the PDBbind benchmark (v.2006). Based on the implemented algorithm, the binding affinity of the identified neutralizing peptide–antigen complexes was calculated as discussed by Karlov et al. (50) and Varela-rial et al. (51).

### Additional computational predictions

The identified peptides were subjected to alanine scanning using Bude Alanine Scan^2^ (52, 53) and Robetta Alanine scan^3^ (54) webservers to infer the energetically significant amino acids at the peptide–antigen interface. This prediction helps to prioritize key residues in the identified peptides. Toxicity and physico-chemical properties of the peptides were predicted using ToxinPred^4^ (55) and the peptide analyzing tool provided by Thermo-fisher Scientific^5^.

## Results

### Neutralizing peptides derived from the CD4-binding site-directed neutralizing antibodies

**FIGURE 2:**
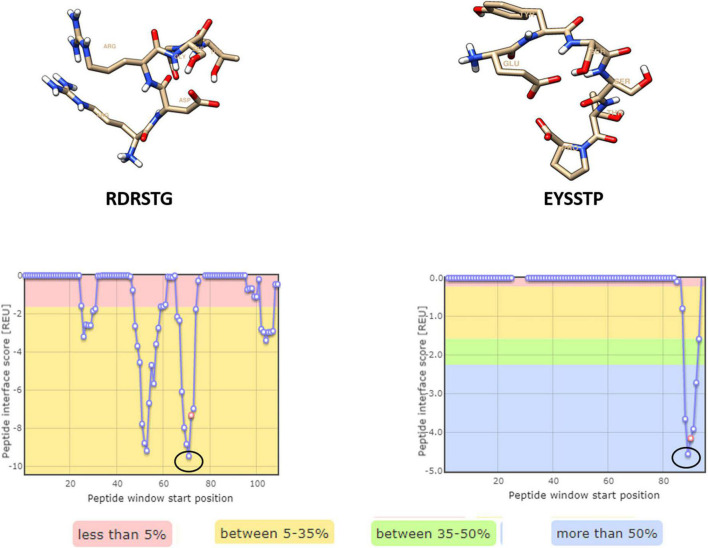
Peptides derived from 8ANC131 with the predicted hotspot regions on the antigen-antibody interface. The regions from where the peptides are derived are highlighted. Peptides were visualized in Chimera, version 1.16.

**FIGURE 3:**
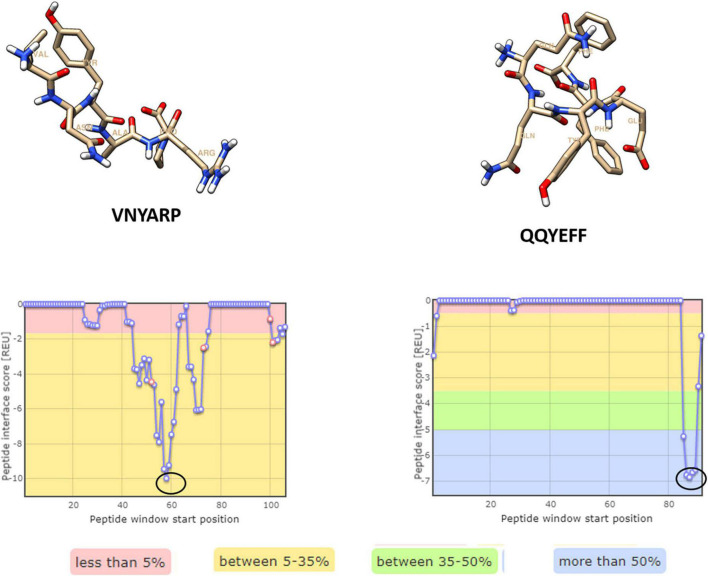
Peptides derived from VRC01 with the predicted hotspot regions on the antigen–antibody interface. The regions from where the peptides are derived are highlighted in a circle [Peptides were visualized in Chimera, version 1.16].

Four hexameric peptides were derived through structure-based sequence inference from the 8ANC131 and VRC01 neutralizing antibody-HIV-1 gp120 complexes using the Rosetta peptiderive protocol as shown in Figures 2, 3. From the hot segments in the bNAbs (that contribute to the most significant binding interaction with the HIV-1 envelope gp120 protein), two peptides were identified from each of the two antigen–antibody complexes. These included the peptide Arg-Asp-Arg-Ser-Thr-Gly (RDRSTG) from the H chain of 8ANC131, which had an interface score of –9.447 and contributed to 29% of binding energy, and the peptide Glu-Tyr-Ser-Ser-Thr-Pro (EYSSTP) from the L chain, which had an interface score of –4.554 and contributed to 101% of binding energy. Two other hexamers were derived from the PDB crystal structure of VRC01-HIV-1C envelope, namely, Val-Asn-Tyr-Ala-Arg-Pro (VNYARP) from the H chain, which had an interface score of –9.982 and contributed to 30% of binding energy, and QQYEFF (Gln-Gln-Tyr-Glu-Phe-Phe) from the L chain, which had an interface score of –6.839 and contributed to 68% of binding energy (Table 1). In general, the peptides derived from the heavy chain of the antibodies gave comparatively lower interface scores than peptides derived from the light chain, signifying better binding affinity of the former. Among the four peptides, RDRSTG peptide having an interface score of –9.447 showed the most significant binding to the HIV-1 envelope.

**TABLE 1:** Peptides derived from neutralizing antibodies and their interface scores.

PDB ID (Co-crystal structure)	Peptide sequence	Receptor (Envelope gp120)	Antibody chain (H-Heavy/ L-Light)	Position in neutralizing antibody (Crystal structure)	Interface score	Total interface score (REU)	Relative interface score (%)
4RWY (8ANC131- subtype B gp120)	RDRSTG	A	H	71–76	–9.447	–33.11	28.54
	EYSSTP	A	L	90–95	–4.554	–4.49	101.32
4LST (VRC01- subtype C gp120)	VNYARP	G	H	57–62	–9.982	–33.57	29.73
	QQYEFF	G	L	89–91, 96–98	–6.839	–10.03	68.23

*Highlighted peptides contribute significantly to binding with the respective antigen.

### Molecular docking of peptides with antigens

**FIGURE 4:**
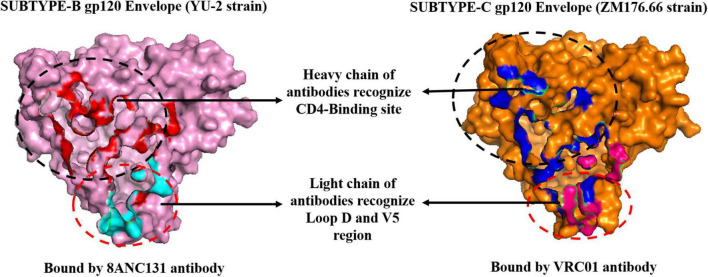
Binding pockets of 8ANC131 and VRC01 in HIV-1 subtype B and subtype C gp120.

**FIGURE 5:**
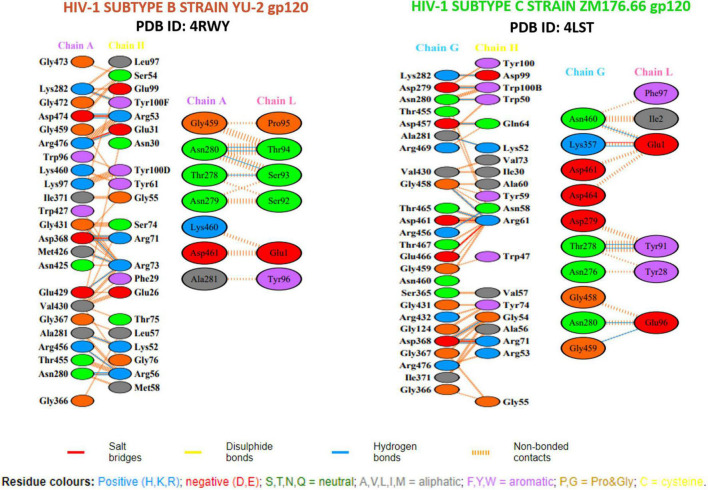
Hydrogen bonding (HB) interactions plot of the crystal structure of antigen–neutralizing antibody complexes.

Structural analysis of the 8ANC131-subtype B gp120 (PDB ID: 4RWY) and VRC01-subtype C gp120 (PDB ID: 4LST) complexes revealed close interaction between the antibody Heavy chains and the HIV-1 gp120 CD4-binding site, while the light chains protruded beyond the CD4bs, particularly the D Loop and V5 regions (Figure 4). Therefore, we excluded the peptides derived from the light chains as they did not engage our target, i.e., the CD4bs. Residues 365–371 of HIV-1 gp120 were found to be the key residues involved in making critical contacts with Phe43 and Arg59 residues of the CD4 receptor (56). The VRC01 antibody showed a non-bonded interaction with Ser365_gp120_, Gly366_gp120_, and Gly367_gp120_ of the Phe43 cavity, while in 8ANC131, Gly366_gp120_ and Gly367_gp120_ were found to be involved in the interaction (57). Furthermore, ASP368_gp120_ was observed to mediate the interaction with ARG71_8*ANC*131_/_*VRC*01_ by forming hydrogen bonds and salt bridges, which mimicked the natural interaction between ARG59_*CD*4 RECEPTOR_ and ASP368_gp120_ (25, 31, 34) (Figure 5).

We also performed intermolecular interaction analysis of the Rosetta-derived peptide-antigen complexes and observed similar interactions as seen in the PDB crystal structures (Supplementary Figure 1). Among the peptides derived from the antibody heavy chains, RDRSTG was found to form two hydrogen bonds with ASP368 (2.75 Å and 2.82 Å) and MET426 (2.72 Å and 3.20 Å), and one hydrogen bond with GLY431 (2.76 Å); the other crystal structure residues were found to have non-bonded contacts in the vicinity of <5 Å (LEU122, VAL430, TRP427 and LYS432). The VNYARP peptide formed two hydrogen bonds with GLY458 (3.08 Å and 3.12 Å) and one hydrogen bond with ARG456 (2.73 Å) and THR467 (3.00 Å). In addition, salt bridges were also observed at ASP461 and GLU466. Other non-bonded contacting residues were ASN280, THR465, GLY366, SER365, ASN460 and ASP457. Based on these observations, we docked the neutralizing antibody 8ANC131-derived peptide RDRSTG with subtype C (ZM176.66) gp120, and the VRC01-derived peptides VNYARP with subtype B (YU-2) gp120, to examine the closeness of the interaction patterns (especially ASP368 and SER365) with that seen in the native crystal structures. To revalidate the observed interactions in the Rosetta derived complexes, we performed re-docking of the peptide RDRSTG with subtype B (YU-2) gp120 and VNYARP with subtype C (ZM176.66) gp120. (Positions of residues are different in PDB crystal structures and 2D LIGPLOT—Supplementary Table 1; Residues stated here are in accordance with the crystal structure but different from that in LIGPLOT).

#### Docking with subtype B gp120

The RDRSTG peptide derived from 8ANC131 was found to form hydrogen bonds with ASP368 (2.96 Å, 2.62 Å), TRP427 (2.81 Å, 2.97 Å), GLY198, GLU370, ASN425, MET426, GLU429 and LYS432, with a binding affinity (Δ*G*) of –9.1 kCal/mol and *K*_*d*_ of 2.2E-07. The peptide VNYARP derived from VRC01 showed hydrogen bond interactions with ASP368 (2.68 Å) and GLY431 (2.86 Å) with a binding affinity (Δ*G*) of –8.6 kCal/mol and *K*_*d*_ of 5.0E-07 (Supplementary Figure 2).

#### Docking with subtype C gp120

The RDRSTG peptide featured interactions at positions SER365 (2.75 Å and 2.69 Å), GLY366, ASP457 (2.78 Å and 2.67 Å), GLY458 and ASN460, with a binding affinity (Δ*G*) of –8.8 kCal/mol and *K*_*d*_ of 3.4E-07. In the case of VNYARP-subtype C gp120 re-docking, hydrogen bond interactions were observed at ASN280 (2.87 Å and 3.10 Å), LYS360, HIS364, ASP457, ASP461 (2.65 Å, 3.23 Å), THR465, GLU466, THR467 and ARG469, thus concurring with the Rosetta peptiderive prediction. However, the main residue SER365 was noticed to form a non-bonded contact with a binding affinity (Δ*G*) of –9.9 kCal/mol and *K*_*d*_ of 5.9E-08 (Supplementary Figure 3). The redocking study demonstrated the predictive accuracy of the methods implemented.

### Molecular dynamics simulation analysis of the peptide-antigen complexes

To start with, the HIV-1 subtype B and subtype C gp120 antigens (without peptides) were subjected to a production run of 200 ns, and trajectory analysis was performed. The system of subtype B gp120 antigen comprised of 49,311 atoms with 14,688 water molecules in the neutralized state, while the subtype C gp120 antigen system comprised of 48,478 atoms with 14,405 water molecules in the neutralized state. The RMSD plot of both antigens revealed that the Cα deviations were stable and within the range of 3 Å, and were found to converge toward the final stages of simulation (Supplementary Figure 4). The RMSF plot identified the peaks which represent the regions/residues that fluctuated the most during the simulation: 175–200 (4.7 Å) and 225–250 (5.0 Å) regions in subtype B gp120, and 250–275 (4.0 Å) and 300–337 (4.2 Å) regions in subtype C gp120 (Supplementary Figure 5).

**FIGURE 6:**
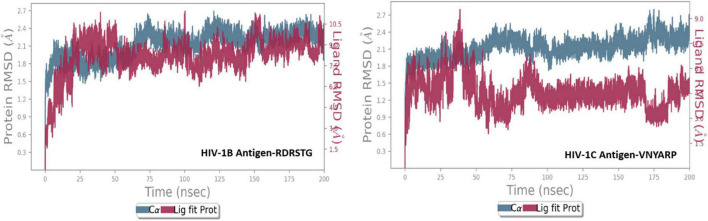
Root mean square deviation (RMSD) plot of the peptide–antigen complexes.

**FIGURE 7:**
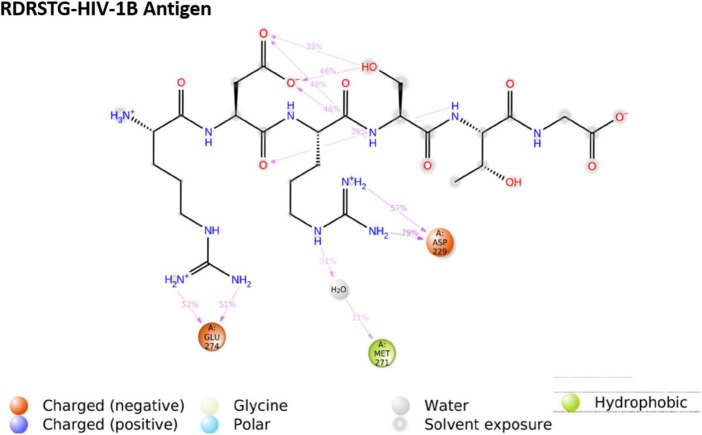
Ligand–protein contacts of peptide Arg-Asp-Arg-Ser-Thr-Gly (RDRSTG) within the subtype B gp120 complex.

We then performed molecular dynamics simulation of the peptide–gp120 complexes. The simulation system of subtype B gp120-RDRSTG solvated complex comprised of 49,305 atoms with 14,654 water molecules, and was neutralized by adding one cl^–^ ion (1.241 mM). On trajectory analysis, the protein–ligand RMSD plot revealed that the complex converged at 10 ns with a 0.6 Å difference between the peptide and antigen-bound state (Figure 6). The ligand RMSD value was in the range of 3.0 Å with reference to the backbone of the antigen and was found to be well-bound to the binding regions. The RMSF plot revealed that RDRSTG (Supplementary Figure 6) interacted well at regions 50–100, 220–250, 250–300 and 300–337, despite fluctuations. Fluctuations posed by the peptide throughout the simulation were inferred from the ligand RMSF plot (Supplementary Figure 7), where it was found to be stable in the range of 4 Å. The structural compactness of the peptide was measured based on the radius of gyration (rGyr). This analysis revealed that the peptide RDRSTG maintained its compactness up to 150 ns in the range of 1 Å (Supplementary Figure 8). The bonded interactions between the antigenic residues and the RDRSTG peptide were analyzed from the ligand–protein contacts plot (Figure 7), wherein it was found that for about 79 and 52% of the duration of the run, Asp229 (ASP368) and Glu274 (GLU429) interacted by means of hydrogen bonds, ionic bonds and water bridges, respectively (Supplementary Figure 10).

**FIGURE 8:**
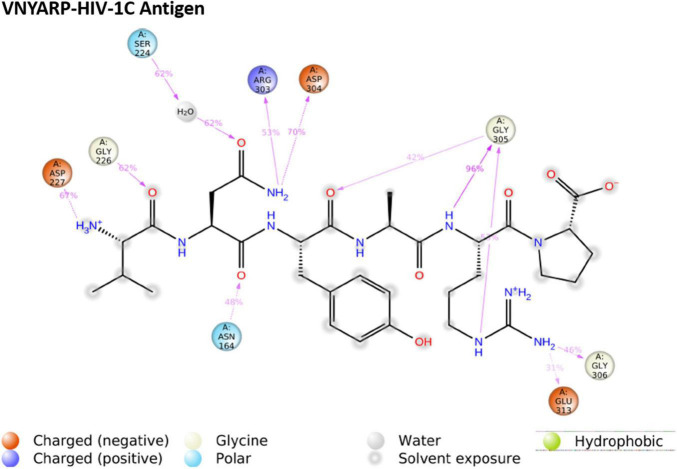
Ligand–protein contacts of peptide Val-Asn-Tyr-Ala-Arg-Pro (VNYARP) within the subtype B gp120 complex.

Subtype C gp120-VNYARP peptide was made up of 48,414 atoms with 14,350 water molecules in the neutralized state. The antigen–peptide RMSD plot inferred that the complex converged at 75 ns with a 0.6 Å difference between the peptide and antigen-bound states (Figure 6). However, the peptide VNYARP evolved to make stable interactions between 85 and 160 ns in the vicinity of <3 Å. The ligand RMSD value was in the range of 2.0 Å with a major fluctuation at 75 ns. Residues in the region 150–180, 200–250, and 300–339 were found to sustain bonded interactions with the peptide as per the RMSF plot (Supplementary Figure 6). The ligand RMSF plot inferred that the peptide is stable as the fluctuations were within the range of 4 Å (Supplementary Figure 7). The rGyr analysis revealed a minimum deviation of 6.0–6.5 Å, indicating that the peptide sustained high compactness during the entire simulation process (Supplementary Figure 9). With regard to peptide–antigen contacts (Figure 8), Gly305 (GLY458) was found to interact 96% of the time during the entire run by means of hydrogen bonds and water bridges. Asp304 (ASP457—70%), Asp227 (ASP368—67%), Gly226 (GLY367—62%) and Ser224 (SER365—62%) formed hydrogen bond interactions and water bridges, with the exception of Asp227 (ASP368), where an additional ionic interaction featured. The least interacting residue was Arg303 (ARG456), which revealed sustained binding (hydrogen bonds and water bridges) around 53% of the 200 ns production run (Supplementary Figure 11).

**FIGURE 9:**
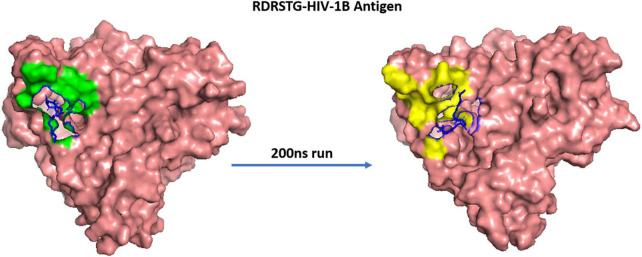
Dynamic evolution of the RDRSTG-HIV-1B gp120 complex. Salmon—HIV-1B YU-2 gp120 envelope; Blue—RDRSTG peptide; shaded regions indicate the interactions before and after simulation.

**FIGURE 10:**
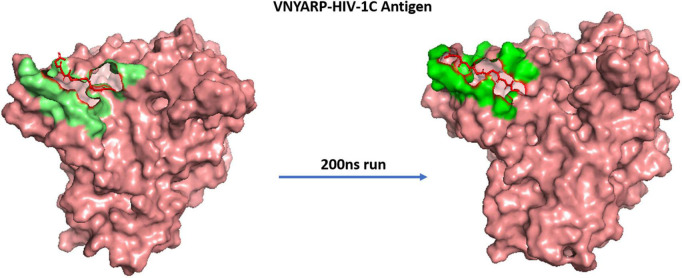
Dynamic evolution of the VNYARP-HIV-1C gp120 complex. Salmon—HIV-1C ZM176.66 gp120 envelope; Red—peptide VNYARP; shaded regions indicate their interactions before and after simulation.

The MD trajectories revealed RDRDTG and VNYARP peptides to be highly stable in terms of bonded interactions during the 200 ns of simulation. The dynamic evolution of the peptides RDRSTG and VNYARP are illustrated in Figures 9, 10. The MD trajectory analyses revealed that the peptides RDRSTG and VNYARP were stable binders, as they feature stable contacts with the key residues namely, SER365, GLY366, GLY367, and ASP368 across the production run (Supplementary Figure 12). The binding free energies (Δ*G*) of the complexes (RDRSTG-subtype B gp120 and VNYARP-subtype C gp120) were calculated over the MD simulation trajectory for the frames sampled at an interval of 20 ns and subjected to MM-PBSA (PB3) using the far-ppi server and binding affinity calculation using Kdeep, respectively. MM-PBSA calculations of RDRSTG-subtype B gp120 and VNYARP-subtype C gp120 complexes gave an average of –13.58 ± 2.85 (Mean ± SD) kCal/mol and –16.04 ± 8.77 (Mean ± SD) kCal/mol, respectively. Similarly, KDeep calculations gave an average of –9.32 ± 0.80 (Mean ± SD) kCal/mol for RDRSTG-subtype B gp120 and –10.18 ± 0.63 (Mean ± SD) kCal/mol for VNYARP-subtype C gp120, respectively (Supplementary Figure 17).

The Alanine scan analysis for RDRSTG-Subtype B gp120 and VNYARP-Subtype C gp120 complexes using Robetta and Bude scan identified the cumulative energetically important amino acids in the peptides across the binding interface as R, D, R, S and T in the RDRSTG peptide and V, N, Y and R in the VNYARP peptide. The binding affinities of the alanine mutated peptides are provided in Supplementary Table 2. The results of the physico-chemical analysis are provided in Table 2. Further, the peptides were found to be non-toxic.

**TABLE 2:** Predicted physico-chemical properties of the neutralizing antibody heavy chain derived peptides.

Peptide	Alanine scan		ToxinPred	Hydrophobicity	Charge	GRAVY	MW Avg. g/mol	MW Mono-isotopic	Theoretical PI
	Bude	Robetta							
RDRSTG	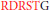	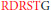	Non-toxic	2.66	+1	–2.40	690.7193	690.3409	10.9
VNYARP	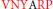	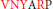	Non-toxic	6.63	+1	–0.82	718.8183	718.3763	9.9

*Highlighted letters indicate hot spot residues in the alanine scan. MW, Molecular weight.

## Discussion

The CD4-binding site of the HIV-1 envelope has been a key target of therapeutics for many years. However, not a single drug targeting the CD4bs has been approved by the US FDA to date (58). With the discovery of broadly neutralizing antibodies, various new approaches have been explored to improve treatment strategies for HIV infection. Despite advancements witnessed in the treatment of HIV, the development of immune therapeutics still remains a cumbersome, time-consuming, and highly expensive process. In recent decades, peptide therapeutics have gained significance in the field of medicine, for being highly specific and efficacious, with good tolerability and safety profiles (59). The interest in peptide therapeutics has been mitigated by certain limitations; these include the relatively short half-life, physiological instability, and difficulty in oral administration (60). However, there have been ongoing efforts to eliminate the obstacles in utilizing peptides, through half-life extension and stability enhancement under physiological conditions (61). Numerous studies have demonstrated the usefulness of short inhibitory peptides in the treatment of several diseases, particularly cancer (62–65). More recently, peptide therapeutics have also shown promise for the treatment of HIV infection (12, 16).

A number of studies in the past have attempted to identify potent peptide inhibitors targeting the CD4bs (29, 66–70), but without much success. This is because a successful inhibitor should not only block the binding of the HIV envelope to the CD4 receptor but should also efficiently block co-receptor interaction which is important for HIV-1 entry into the target cell (71). This kind of inhibition is actually accomplished very well by neutralizing antibodies, which target specific epitopes on the virus and lead to virus neutralization, thereby preventing HIV infection. Modern methods in computer-aided drug design have catalyzed the ability to reduce cost and time which limits the development of novel therapeutics (72).

Andrianov et al. (73) utilized a computer-aided strategy to screen a public web-oriented virtual screening platform (pepMMsMIMIC) to identify a few promising peptidomimetic candidates from the broadly neutralizing antibody VRC01 (73). In a similar line, we undertook an in-depth analysis of the co-crystal structures of the bNAb 8ANC131-subtype B YU-2 gp120 and VRC01-subtype C ZM176.66 gp120 complexes and inferred that the contacts made by each CD4bs-directed broadly neutralizing antibody with the HIV-1 gp120 were highly variable. However, it was observed that the heavy chain of the CD4bs-directed neutralizing antibodies engaged well with the CD4bs, i.e., the Phe43 cavity, which is highly conserved among the different bNAbs. Based on earlier studies as well as our analysis of the co-crystal structures of the antibody-antigen complexes, we decided to narrow down on hexameric peptides that would be short and at the same time target the critical residues in the CD4bs. Subsequently, potential hexamers were derived from the crystal structures of 8ANC131-subtype B gp120 and VRC01-subtype C gp120. Two peptides were predicted from each crystal structure, one from the heavy chain and another from the light chain. Only the peptides derived from heavy chains were taken up for further computational evaluations as they bound best to the CD4bs. The heavy chain derived peptides were docked with subtype B and subtype C envelopes, to identify interactions with the key residues in the CD4bs. Based on the 2D-interaction plot of the crystallized complexes, peptides RDRSTG and VNYARP, derived from the heavy chain of 8ANC131 and VRC01, respectively, were shortlisted as they interacted with the key residues of the CD4bs mentioned earlier. Molecular dynamics simulation of the RDRSTG-subtype B gp120 and VNYARP-subtype C gp120 complexes across the 200 ns trajectory (frames sampled at an interval of 20 ns) revealed that the peptides RDRSTG and VNYARP precisely target the binding site of the CD4 receptor (Phe43 and Arg59 contacts) and interact with the critical residues through hydrogen bonds and Vander Waal’s interactions with an average binding free energy (Δ*G*) (MM-PBSA) of –13.58 ± 2.85 (Mean ± SD) kCal/mol and –16.04 ± 8.77 (Mean ± SD) kCal/mol, respectively. The sampled frames were also subjected to KDeep calculation, wherein, the peptides RDRSTG and VNYARP scored a significant average binding affinity (Δ*G*) of –9.32 ± 0.80 (Mean ± SD) kCal/mol and –10.18 ± 0.63 (Mean ± SD) kCal/mol, respectively. In the case of VNYARP, one of the frames at the 60th ns gave a higher MMPBSA value (Δ*G* = +3.18 kCal/mol) due to a major conformation change; however, the lower binding free energy state was quickly regained around the 80th ns.

The energetically significant amino acids in the topmost stable peptide-antigen complexes of RDRSTG-subtype B gp120 and VNYARP-subtype C gp120 were found to be R, D, R, S, T, V, N, Y and R, as inferred from the cumulative results of the alanine scan (52, 53) and Robetta analyses (54, 74). The two peptides were also predicted to possess favorable physicochemical properties including non-toxicity, hydrophobicity of 2.66 and 6.63, and GRAVY (Grand Average of Hydropathy) of –2.40 and –0.82 (75, 76), respectively (Table 2) making these highly promising therapeutic candidates. A striking finding to be noted is that the RDRSTG peptide is derived from the site that is involved in the critical interaction between ARG59_CD4–RECEPTOR_ and ASP368_gp120_. This could be the likely reason for this peptide standing out as the best CD4bs-targeting neutralizing peptide, as compared to all other peptides.

We further analyzed the co-crystal structures of other VH-gene-restricted (VRC01-class and 8ANC131-class) and CDR-H3-dominated antibodies with gp120 envelope for their residual interactions. The VRC01-class antibodies 3BNC117 (PDB ID: 4JPV), N6 (PDB ID: 5TE7) and NIH45-46 Fab (PDB ID: 4JDV) revealed interactions between the conserved ARG71_HC_/_Heavy Chain_ residue and ASP368_gp120_. In addition, these antibodies also interacted with SER365_gp120_, GLY366_gp120_ and ASP368_gp120_ through Leu44_CD4_ and Lys46_CD4_ (10, 57, 77). In case of 8ANC131-class antibodies (1B2530; PDB ID: 4YFL) and CDR-H3 dominated antibody (CH103; PDB ID: 4JAN), the key contacts were ASP368_gp120_ through ARG72_HC_ and ARG97_HC_, respectively. These antibodies also showed interaction with residues of the PHE43 cavity in gp120 (34, 78). Given these observations, we speculate that peptides derived from these neutralizing antibodies could also be explored for the identification of novel neutralizing peptide mimetics against HIV.

The binding of HIV-1 gp120 with the CD4 receptor on the target cell triggers a conformational change that uncovers epitopes called CD4-induced (CD4i) epitopes that bind to the chemokine co-receptors on the host cell, either CCR5 or CXCR4. Since the binding of the candidate bNAb mimetics to the CD4bs prevents conformational changes in the HIV-1 gp120 and obsoletes binding to the co-receptor, the process of viral entry into the target cells is also inhibited. Thus, the peptide mimetics identified in this study hold promise as highly potent candidates for HIV therapeutics.

## Conclusion

Using modern computational tools the present study identified two short, hexameric peptides from the heavy chain of two well-characterized CD4bs-targeting bNAbs, 8ANC131 and VRC01, that hold promise as potential therapeutic candidates that can be exploited for the treatment of HIV-infected persons. This study is the first of its kind to identify short peptides that can bind to and possibly neutralize HIV-1. Given the potential of the identified candidate peptides to function as mimetics of HIV-1 broadly neutralizing antibodies, *in vitro* studies are in progress to validate their efficacy in HIV-1 neutralization in our laboratory (20).

## Data availability statement

The original analyses presented in this study are included in the article/Supplementary material. Further inquiries can be directed to the corresponding authors.

## Author contributions

UV and LH: conceptualization, resources, writing—review and editing, and supervision. SV: methodology, formal analysis, investigation, data curration, and writing—original draft preparation. SV, UV, and LH: validation. LH: project administration. All the authors have read and agreed to the published version of the manuscript.

## Footnotes

1
https://www.unaids.org/en

2
https://pragmaticproteindesign.bio.ed.ac.uk/balas/

3
https://robetta.bakerlab.org/queue.jsp

4
http://crdd.osdd.net/raghava/toxinpred/

5
https://www.thermofisher.com/in/en/home/life-science/protein-biology/peptides-proteins/custom-peptide-synthesis-services/peptide-analyzing-tool.html
