# Supplementary material for: Design of human immunodeficiency virus-1 neutralizing peptides targeting CD4-binding site: An integrative computational biologics approach
Source: Front Med (Lausanne). 2022 Nov 18;9:1036874. doi: 10.3389/fmed.2022.1036874 (PMC9715589; doi:10.3389/fmed.2022.1036874)
Supplement: Supplementary file 1 [file Data_Sheet_1.pdf]

SUPPLEMENTARY CONTENTS

TABULAR COLUMNS

Table S1. Position of amino acids in the CD4bs of PDB crystal structures and Rosetta-derived complexes

Amino acid (Three letter code) Subtype B YU-2 strain	Position in PDB crystal structure	Position in Rosetta derived complex	Amino acid (Three letter code) Subtype C ZM176.66 strain	Position in PDB crystal structure	Position in Rosetta derived complex
Leu	122	79	Asn	280	164
Thr	123	80	Ala	281	165
Gly	124	81	Lys	360	219
Gly	198	82	Glu	362	221
Asp	368	229	His	364	223
Glu	370	231	Ser	365	224
Asn	425	270	Gly	366	225
Met	426	271	Gly	367	226
Trp	427	272	Asp	368	227
Gln	428	273	Thr	455	302
Glu	429	274	Arg	456	303
Val	430	275	Asp	457	304
Gly	431	276	Gly	458	305
Lys	432	277	Gly	459	306
Gly	473	318	Asn	460	307
Asp	474	319	Asp	461	308
			Asp	462	309
			Thr	465	312
			Glu	466	313
			Thr	467	314
			Arg	469	316

Table S2. Binding affinities ΔΔG (KJ/mol) of Alanine mutated peptides

**RDRSTG-Subtype B antigen**				**VNYARP-Subtype C antigen**			
**Robetta**	**ΔΔG (KJ/mol)**	**Bude**	**ΔΔG (KJ/mol)**	**Robetta**	**ΔΔG (KJ/mol)**	**Bude**	**ΔΔG (KJ/mol)**
Arg (R)	1.70	Arg (R)	2.1	Val (V)	0.56	Val (V)	1.6
Asp (D)	1.44	Asp (D)	3.3	Asn (N)	2.98	Asn (N)	6.6
Arg (R)	1.86	Arg (R)	7.6	Tyr (Y)	0.47	Tyr (Y)	2.5
Ser (S)	0.05	Ser (S)	2.4	Arg (R)	2.46	Arg (R)	11.3
Thr (T)	0.39	Thr (T)	0.7			Pro (P)	1.9

FIGURES

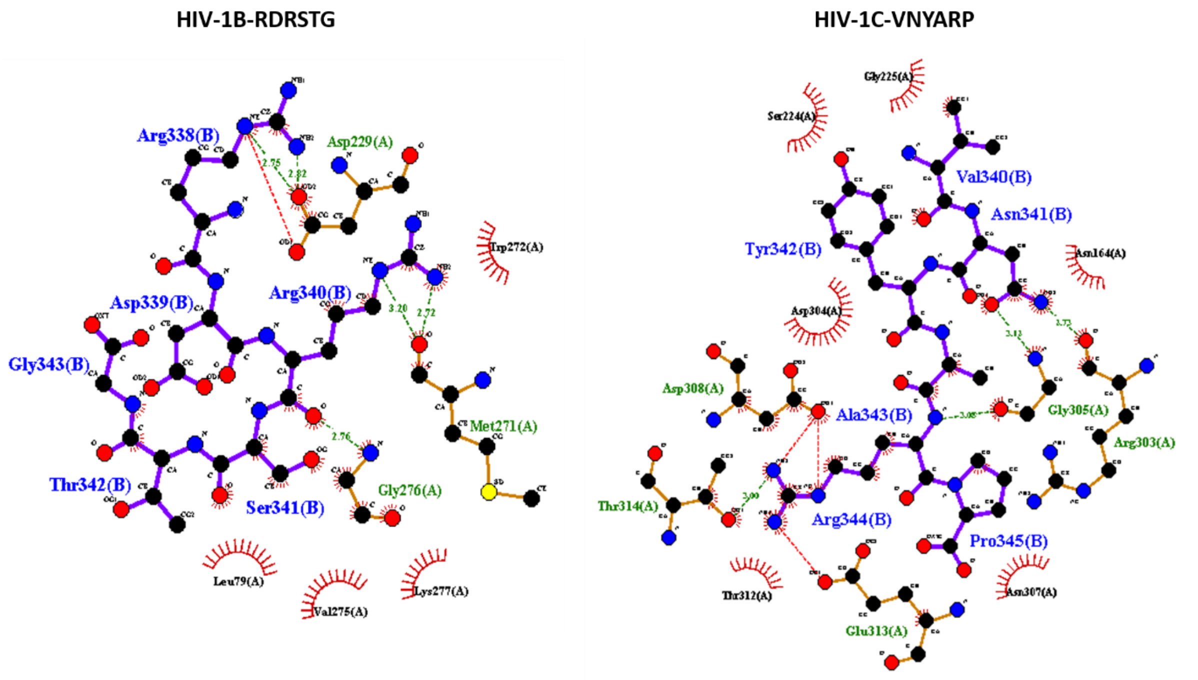

Figure S1. LIGPLOT of Rosetta derived antigen-peptide complexes (Before simulation)

Chain A- HIV-1 gp120 envelope; Chain B-peptides (peptide numbering: RDRSTG- 338 to 343; VNYARP- 340 to 345 as given by Rosetta pepti-derive server)

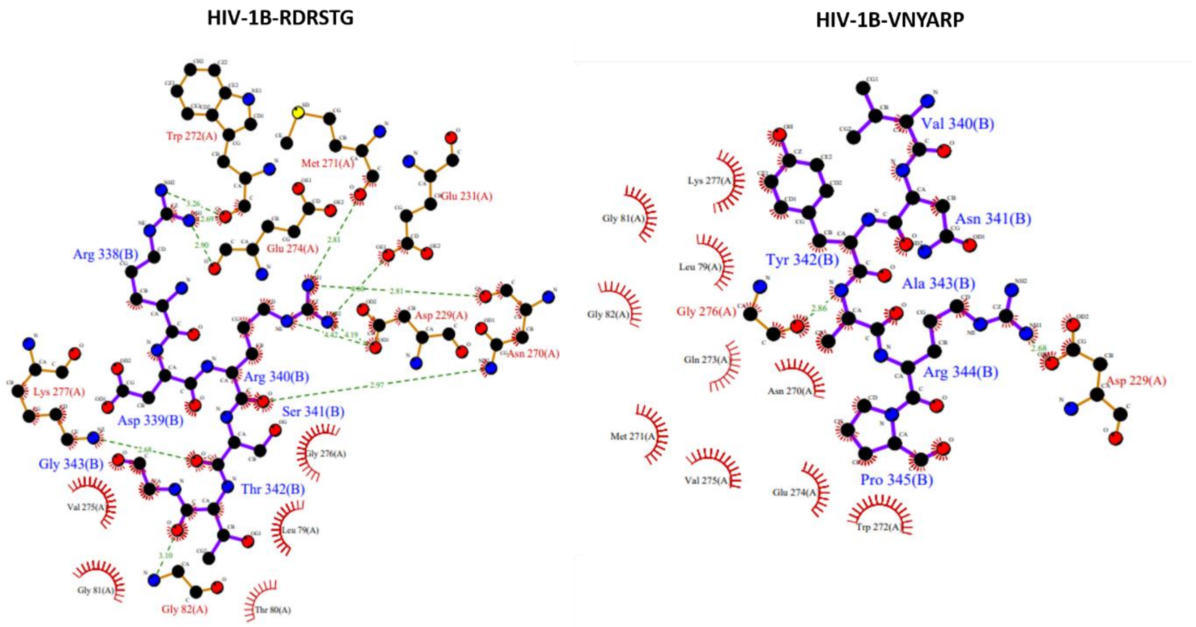

Figure S2. LIGPLOT of docking interactions between HIV-1B gp120 and peptides

Chain A- HIV-1 gp120 envelope; Chain B-peptides (peptide numbering: RDRSTG- 338 to 343; VNYARP- 340 to 345 as given by Rosetta pepti-derive server)

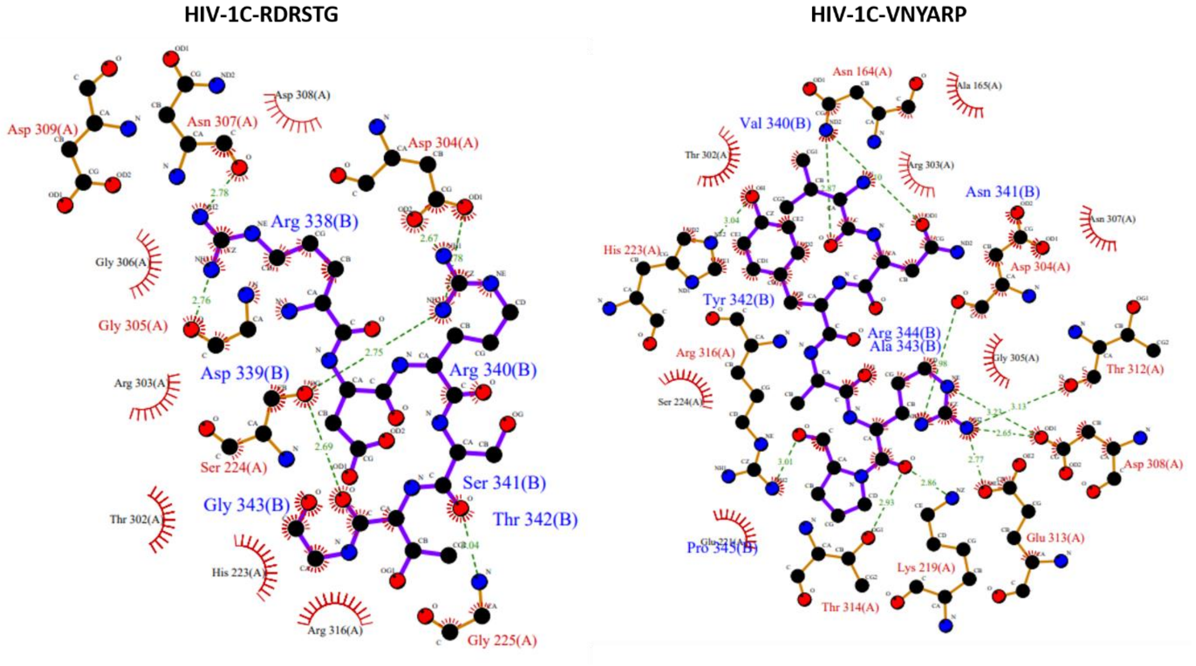

Figure S3. LIGPLOT of docking interactions between HIV-1C gp120 and peptides

Chain A- HIV-1 gp120 envelope; Chain B-peptides (peptide numbering: RDRSTG- 338 to 343; VNYARP- 340 to 345 as given by Rosetta pepti-derive server)

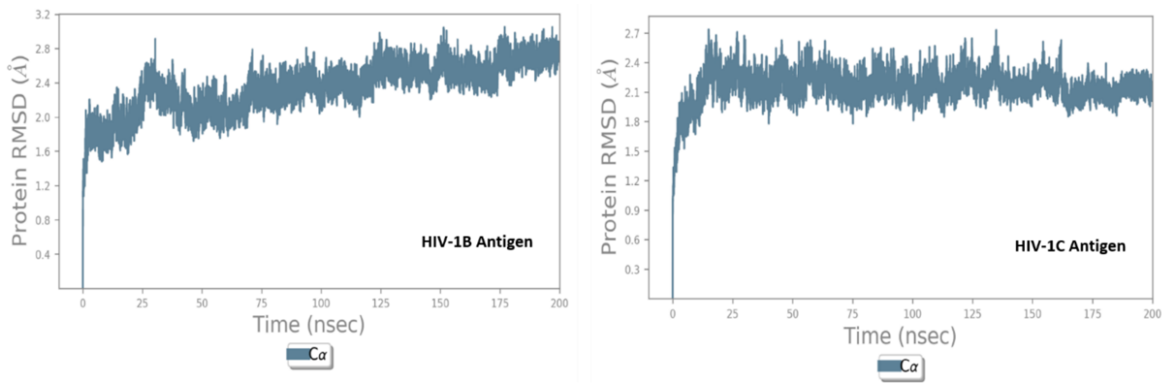

Figure S4. Root Mean Square Deviation (RMSD) plot of subtype B and C gp120 antigen

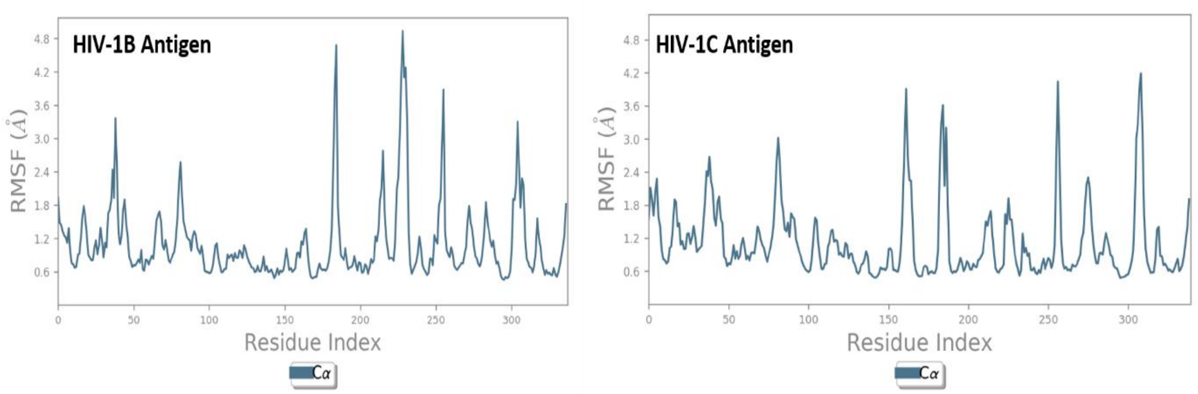

Figure S5. Root Mean Square Fluctuations (RMSF) plot of subtype B and C gp120 antigen

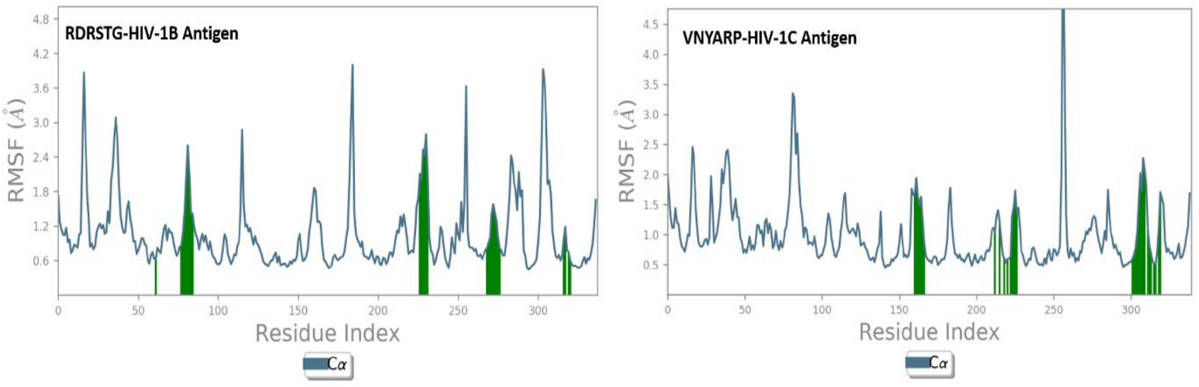

Figure S6. Root Mean Square Fluctuations (RMSF) plot of antigen-peptide complexes

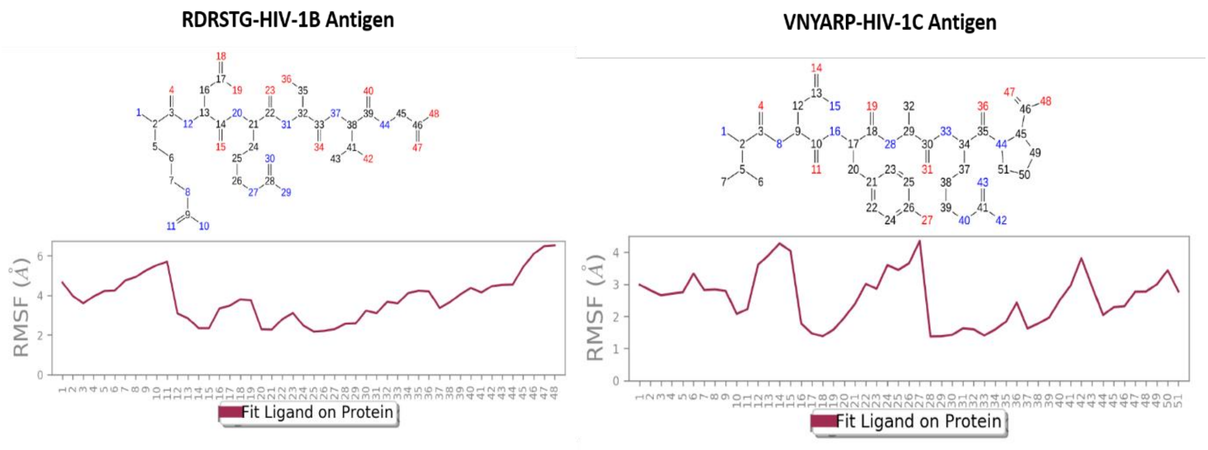

Figure S7. Root Mean Square Fluctuations (RMSF) plot of peptides (Ligand RMSF)

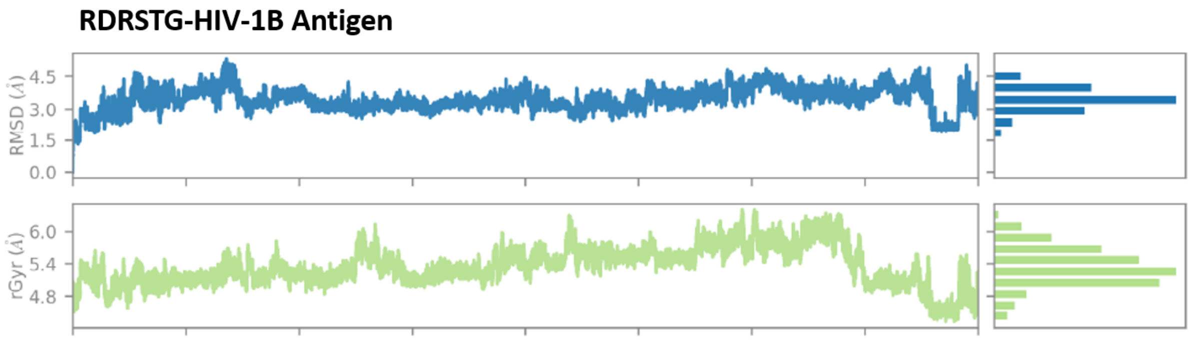

Figure S8: Ligand properties of RDRSTG with subtype B gp120

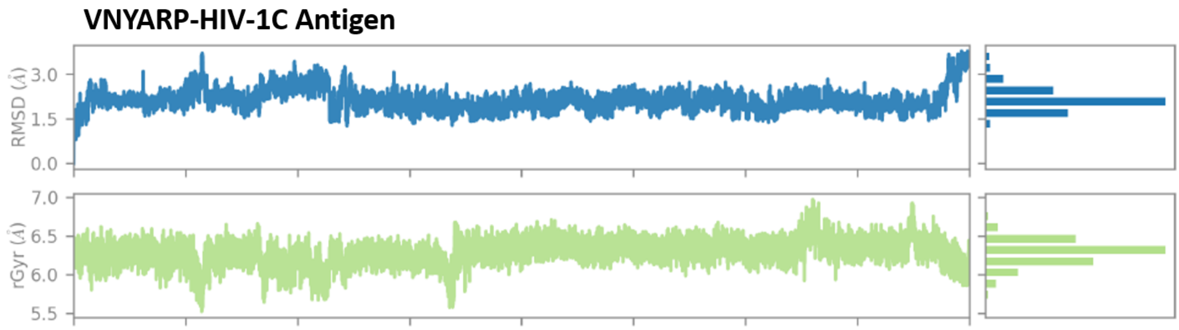

Figure S9: Ligand properties of VNYARP with subtype C gp120

RDRSTG-HIV-1B Antigen

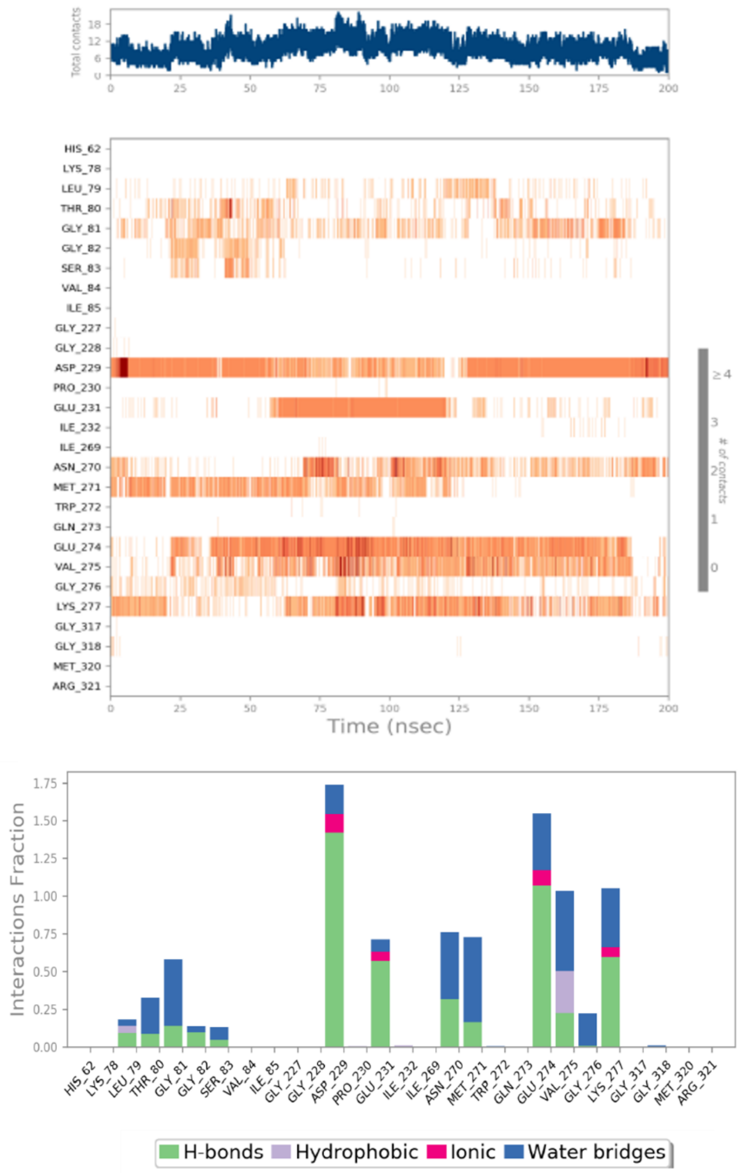

Figure S10. Protein-ligand contacts of RDRSTG with subtype B gp120

VNYARP-HIV-1C Antigen

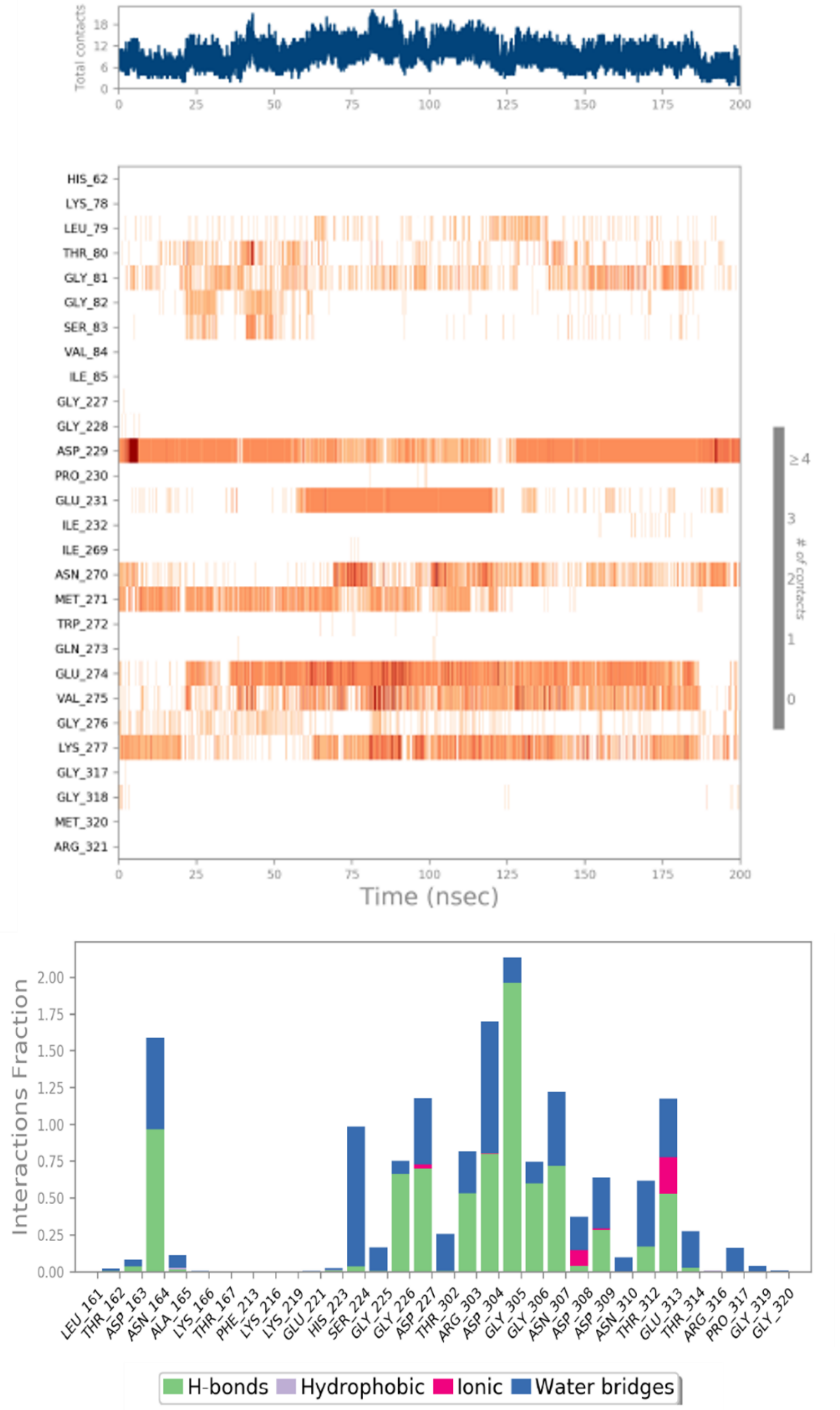

Figure S11. Protein-ligand contacts of VNYARP with subtype C gp120

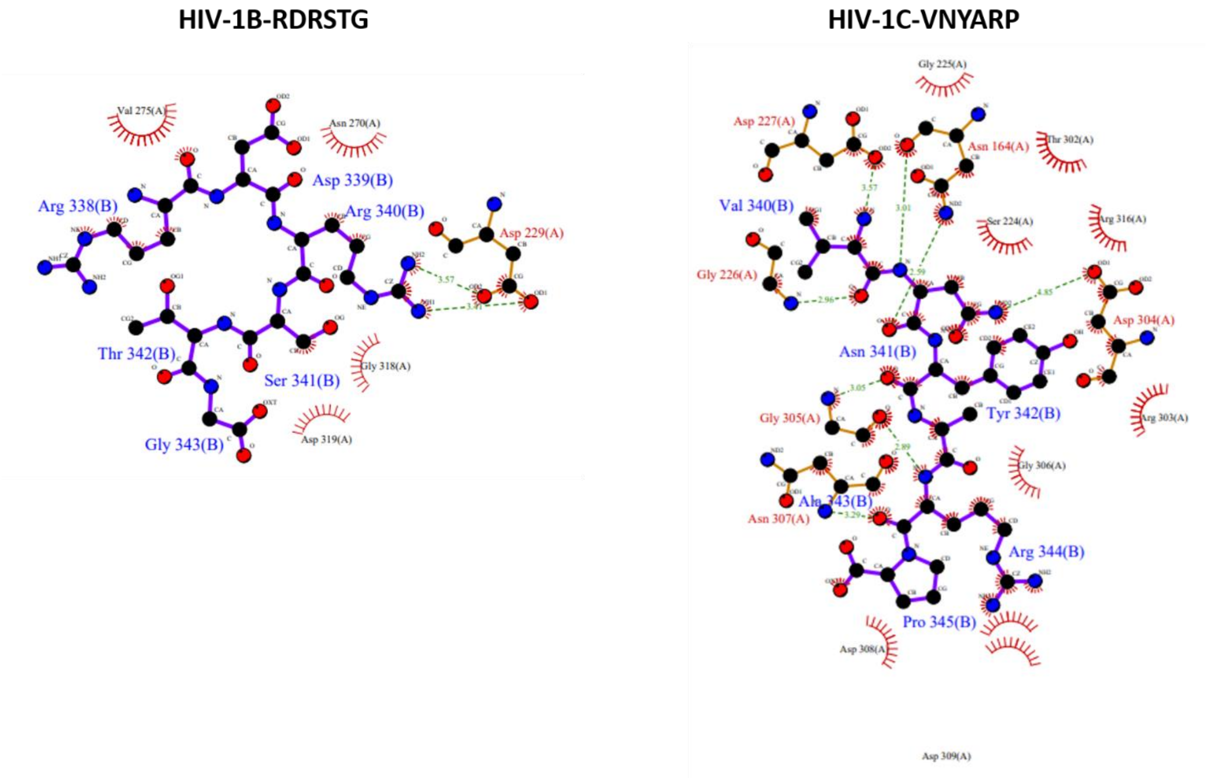

Figure S12. LIGPLOT of peptides at the end of MD simulation for 200ns (Final frame of the complexes)

Chain A- HIV-1 gp120 envelope; Chain B-peptides (peptide numbering: RDRSTG- 338 to 343; VNYARP- 340 to 345 as given by Rosetta pepti-derive server)

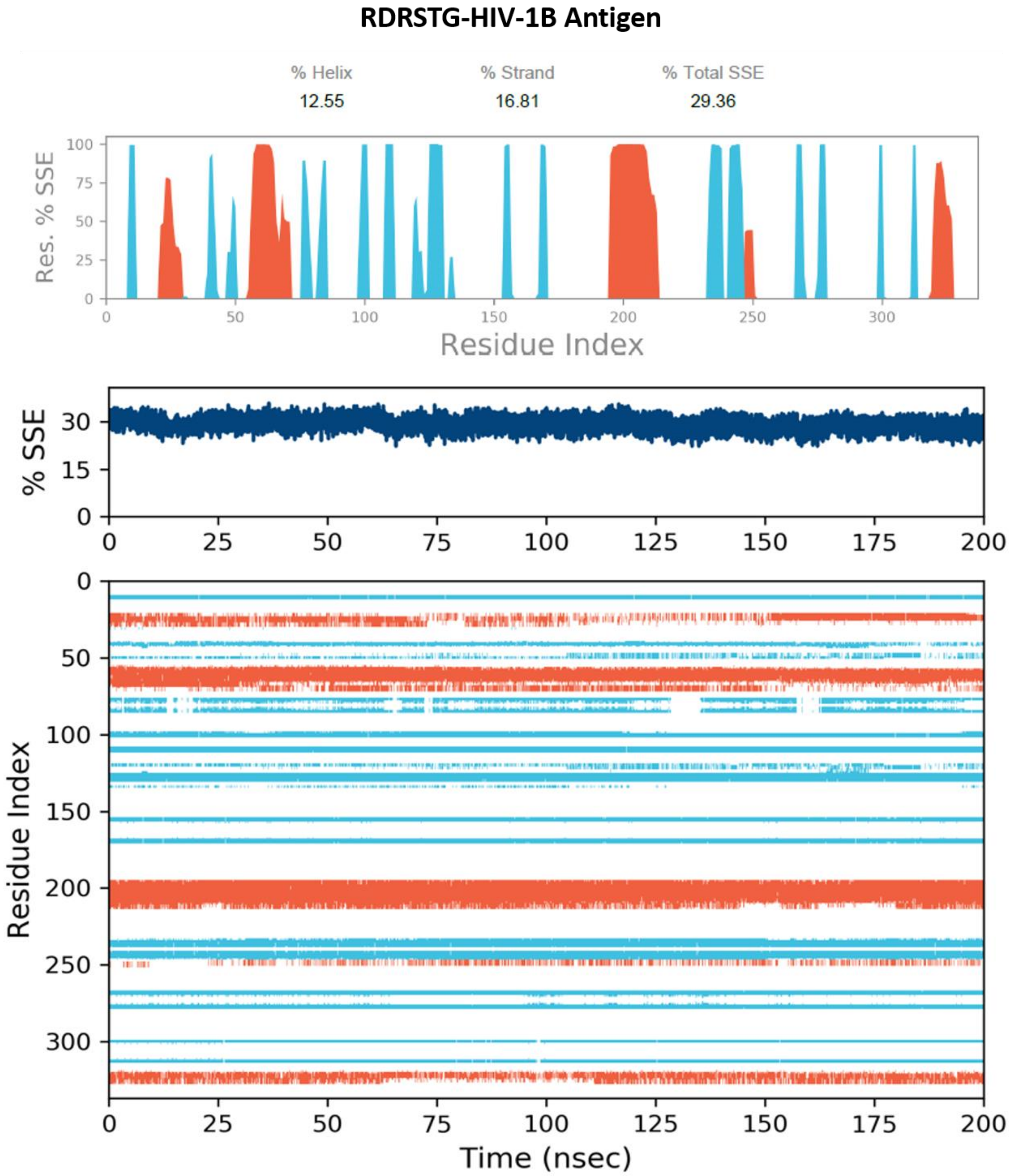

Figure S13. Plot showing RDRSTG-Subtype B gp120 Secondary Structure Elements (SSEs) distribution by residue index, SSE composition over the trajectory frames and its assignment over the course of 200ns simulation

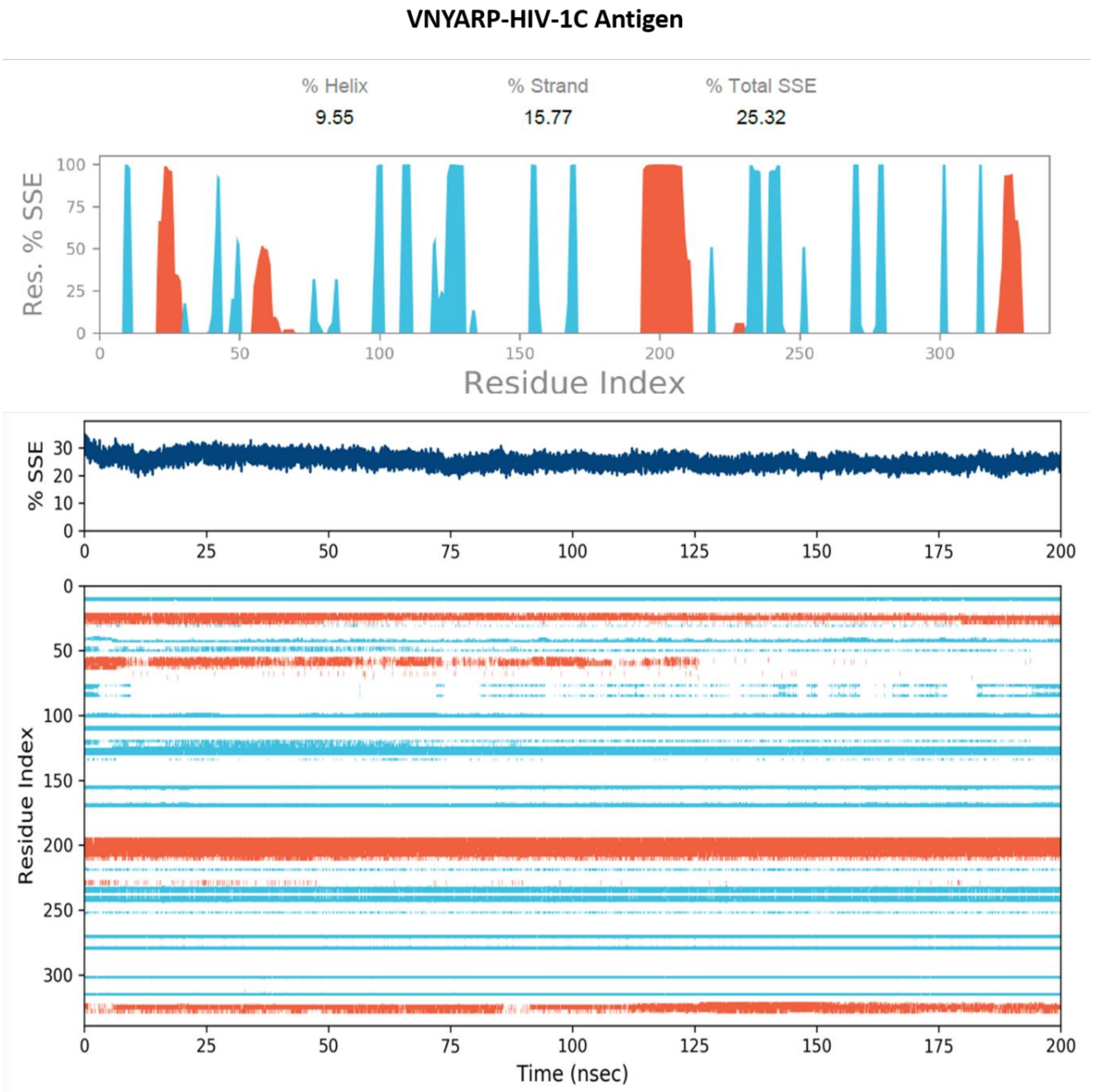

Figure S14. Plot showing VNYARP-Subtype C gp120 Secondary Structure Elements (SSEs) distribution by residue index, SSE composition over the trajectory frames and its assignment over the course of 200ns simulation

RDRSTG-HIV-1B Antigen

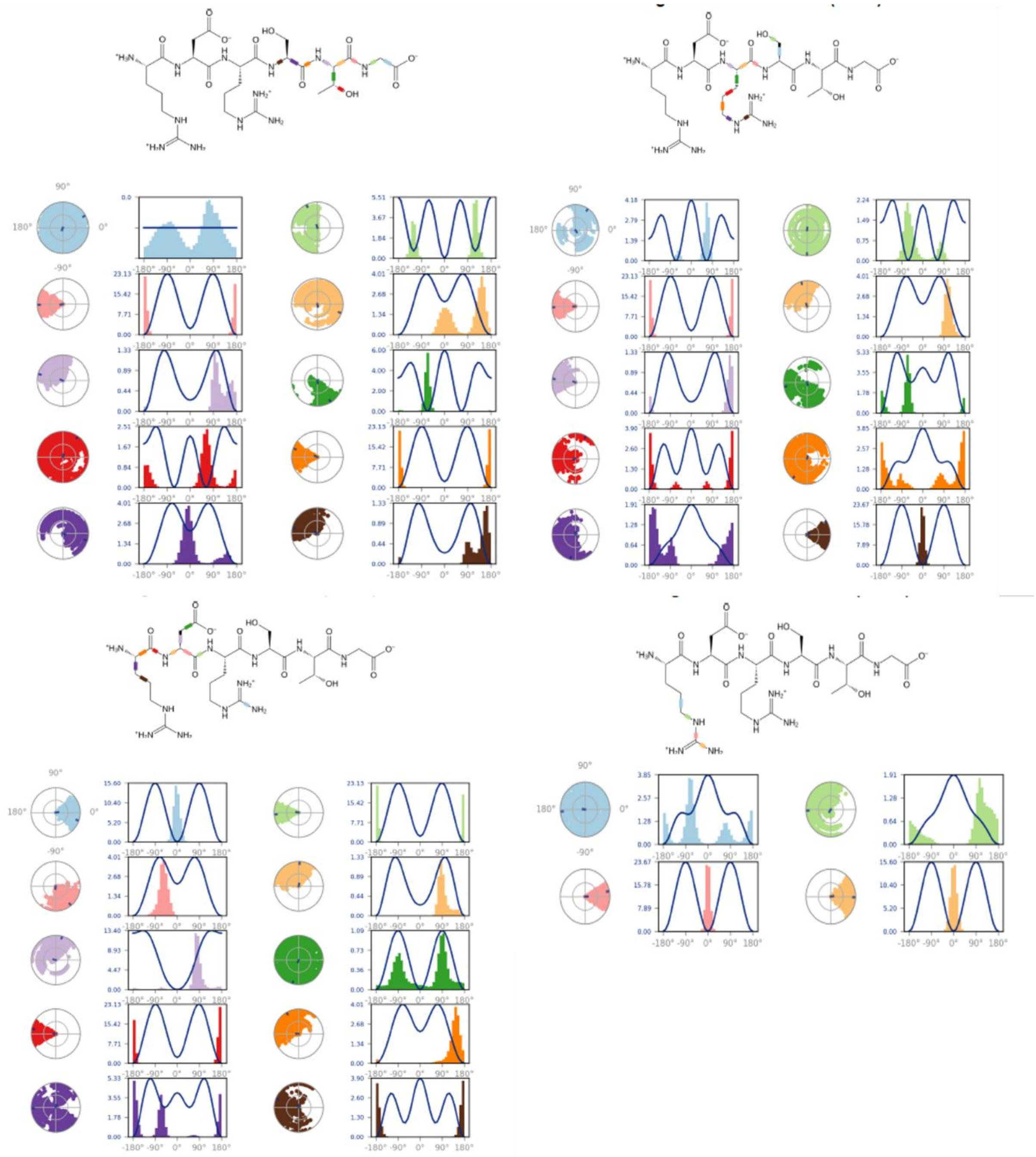

Figure S15. Ligand Torsion plot depicting the conformational evolution of every Rotatable Bond (RB) in RDRSTG across the simulation trajectories

VNYARP-HIV-1C Antigen

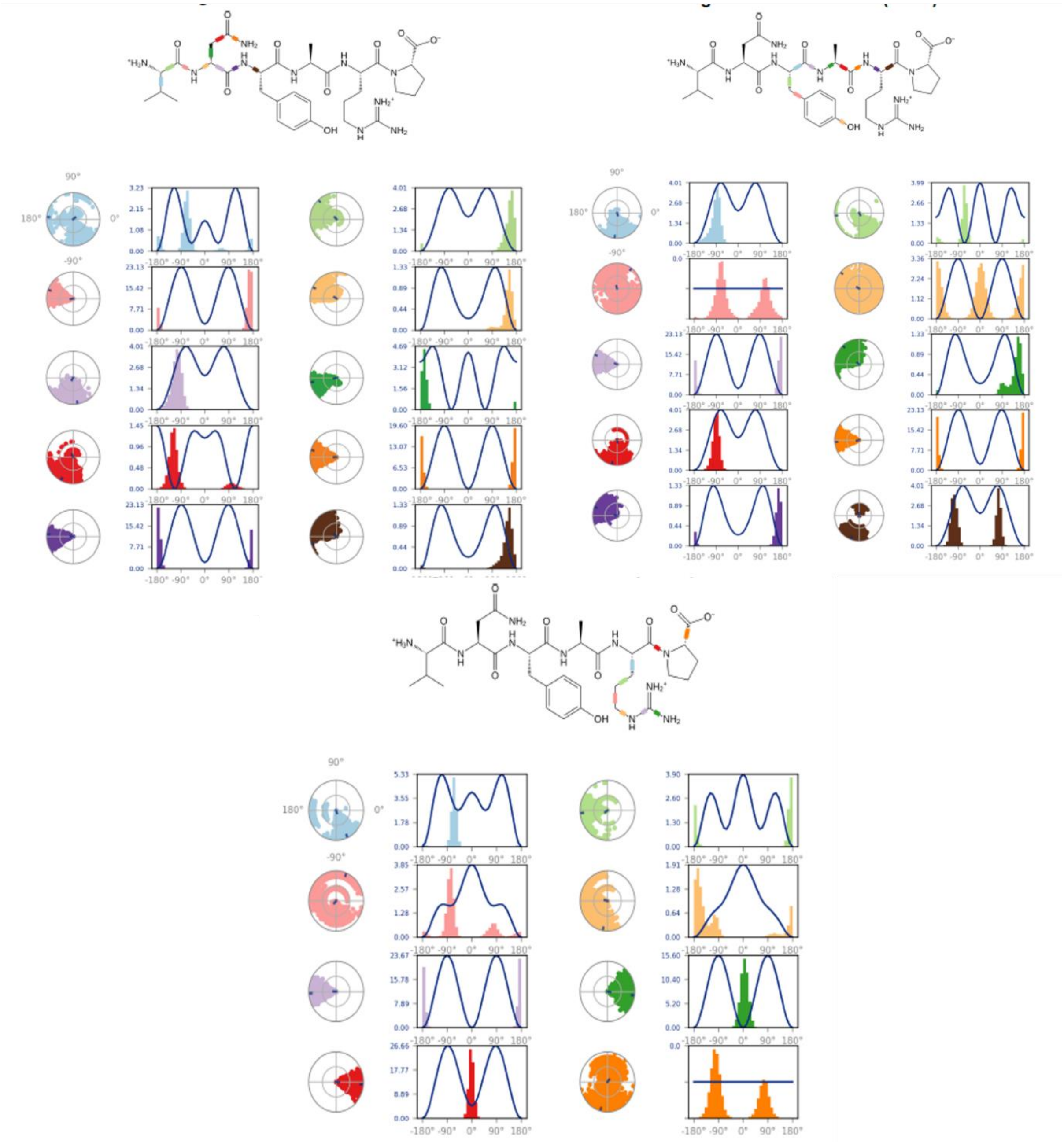

Figure S16. Ligand Torsion plot depicting the conformational evolution of every Rotatable Bond (RB) in VNYARP across the simulation trajectories

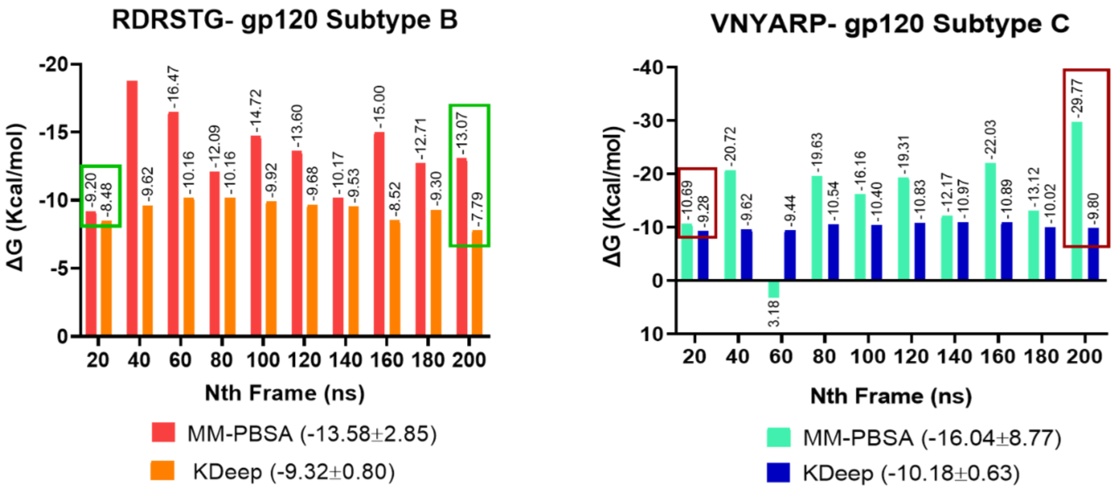

Figure S17. Binding free energy (ΔG) of RDRSTG-HIV-1B gp120 complex and VNYARP-HIV-1C gp120 complex during 200ns dynamic simulation with frame intervals of 20ns.
